# Evidence for reversible light-dependent transitions in the photosynthetic pigments of diatoms†

**DOI:** 10.1039/d2ra05284a

**Published:** 2022-11-03

**Authors:** Charalampos Tselios, Constantinos Varotsis

**Affiliations:** Cyprus University of Technology, Department of Chemical Engineering Lemesos Cyprus c.varotsis@cut.ac.cy

## Abstract

Marine diatoms contribute to oxygenic photosynthesis and carbon fixation and handle large changes under variable light intensity on a regular basis. The unique light-harvesting apparatus of diatoms are the fucoxanthin–chlorophyll *a*/*c*-binding proteins (FCPs). Here, we show the enhancement of chlorophyll *a*/*c* (Chl *a*/*c*), fucoxanthin (Fx), and diadinoxanthin (Dd) marker bands in the Raman spectra of the centric diatom *T. pseudonana*, which allows distinction of the pigment content in the cells grown under low- (LL) and high-light (HL) intensity at room temperature. Reversible LL–HL dependent conformations of Chl *c*, characteristic of two conformations of the porphyrin macrocycle, and the presence of five- and six-coordinated Chl *a*/*c* with weak axial ligands are observed in the Raman data. Under HL the energy transfer from Chl *c* to Chl *a* is reduced and that from the red-shifted Fxs is minimal. Therefore, Chl *c* and the blue-shifted Fxs are the only contributors to the energy transfer pathways under HL and the blue- to red-shifted Fxs energy transfer pathway characteristic of the LL is inactive. The results indicate that *T. pseudonana* can redirect its function from light harvesting to energy-quenching state, and reversibly to light-harvesting upon subsequent illumination to LL by reproducing the red-shifted Fxs and decrease the number of Dds. The LL to HL reversible transitions are accompanied by structural modifications of Chl *a*/*c* and the lack of the red-shifted Fxs.

## Abstract 2

A reversible light-intensity behavior of Dds and Fxs composition in the cells of *T. pseudonana*. The observed LL to HL reversible transitions are accompanied by structural modifications of Chls *a*/*c* and the lack of the red-shifted Fxs.

## Graphical abstract

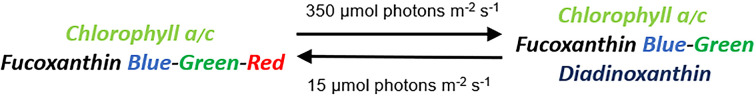

## 1. Introduction

Photosynthetic organisms contain light-harvesting antenna systems exposed to varying irradiation for gathering light energy necessary for driving photochemical reactions.^1–8^ Diatoms are a group of microalgae found in oceans and fresh water and responsible for fixing CO_2_ into organic carbon and because of their silicified cell wall have emerged as major drivers in the silicon cycle.^2,3^ The whole genomes of two diatom species, *Phaeodactylum tricornutum* and *Thalassiosira pseudonana* (*T. pseudonana*) were sequenced, and a few analogues of the genes that occur in plants or algae were found in these diatoms.^9,10^ The photoacclimation strategy of species growing under variable light conditions enables the efficient regulation of photosystem structures to the amount of absorbed energy.^11^ The spectral composition and energy of light plays a crucial role in the rate of growth, the photosynthetic efficiency and photoprotective mechanisms, and thereby in pigment content which is similar but with variable ratio between them in each diatom species.^11,12^ The genomes of diatoms contain a large number of *Lhc* genes and the unique light-harvesting apparatus of diatoms are fucoxanthin–chlorophyll *a*/*c*-binding proteins (FCPs) which are the major contributors to their survival under intense and variable light conditions.^6^

Chl *a*/*c* and fucoxanthin (Fx) absorb light in the blue-green region that is also available under water.^11–13^ In addition to Fx, diadinoxanthin (Dd) and diatoxanthin (Dt) are also present in the FCPs. When light is transiently in excess, induction of nonphotochemical quenching (NPQ) occurs whereby the accumulation of a proton gradient, and consequently ΔpH, across the thylakoid membrane drives the de-epoxidation of Dd pigments, *via* the protection pathway Dd (light harvesting) to Dt (photo-protective).^14–23^ Although, the photosynthetic and photoprotective functions of diatoms and the structural basis for blue-green light harvesting and energy dissipation as well as the contribution of certain pigments have been investigated, a fundamental understanding of the physical mechanisms remains elusive.^4,13,14^ Currently, single-molecular spectroscopy and computational methods have demonstrated how reduced excitonic coupling enhances light harvesting in the main antennae of diatoms.^24,25^ Certain key-issues related to the structure–conformational function relationship of the photosynthetic pigments involved in the light-dependent photo-energy transfer transitions of diatoms at physiological room temperature conditions have not been investigated.^26–30^ Raman spectroscopy is a non-destructive, structure sensitive technique, and has been applied in a variety of biological dynamics studies.^27,28,31–37^ Carotenoids and chlorophylls are exceptional pigments in Raman spectroscopic analysis of photosynthetic communities because they have strong Raman signals and are present in all photosynthetic organisms.^27,33,36,37^ Previous studies have focused on the identification of the isolated carotenoids Fx, Dd and Dt and in intact *Cyclotella meneghiniana* cells under frozen conditions (77 K) with variable excitations from 413.1 nm to 570 nm.^28^ It was concluded that significant conformational differences in Dd and Dt were observed at 77 K between the cells grown under 40 and 140 μmol photons per m^2^ per s conditions.^27,37^ The photodynamic behaviour of the carotenoids under physiological room temperature conditions and their behaviour under high light (HL) intensity cell-growth conditions has not been investigated. In addition, data on the conformational coupled changes of Chl *a*/*c* and carotenoids pigments that provide valuable insights into the energy regulations that safely dissipates excess energy to protect the system against deleterious photoproducts at room temperature are not available. In the work presented here, we have applied a combination of Uv-vis, resonance Raman and fluorescence spectroscopy to investigate the photodynamic behavior and the structure function behavior of Chl *a*/*c* and the carotenoids in the whole cells of *T. pseudonana* grown under 15 μmol photons per m^2^ per s (LL), 150 μmol photons per m^2^ per s (IL) and 350 μmol photons per m^2^ per s (HL) growth culture conditions. This way, we probed the sequential increase of Dd cycle pigments in the membranes and, thus, monitor the interactions and binding sites of the pigments. Based on the frequencies of the CaN bands of Chl *c* at 1361 and 1355 cm^−1^, we suggest that Chl *c* is present in two light dependent reversible conformations and adopts a more planar configuration in the transition from LL to HL cell growth condition. In addition, the coordination marker bands demonstrate the existence of five- and six-coordinated Chl *a*/*c* with weak axial ligands. The LL to HL cell-growth transition is characterized by significant changes in the RR marker bands of Chls *a*/*c*, Fx, and Dd/Dt. In the HL to LL cell-growth transition all pigments have similar characteristics with those detected in the spectra of the LL samples demonstrating in combination with the Uv-vis that the LL–HL transition is reversible. Under HL, the reduced energy transfer from Chl *c* to Chl *a* is attributed to the limited presence of the red-shifted Fxs in the 525–550 nm region that causes modifications of pigment–pigment and/or pigment–protein interactions and in the presence of additional Dd's. The energy transfer from Chls *c* to Chls *a* is accompanied by conformational changes of Chls *a*/*c* that is expected to affect their excitonic coupling. We report that under HL, the red-shifted Fxs contribution to the energy transfer is negligible and the energy transfer pathway from the blue-shifted Fxs to the red-shifted Fxs that is operative under LL conditions does not exist. The observed blue-shifted 439/435 nm and red-shifted 680/683 nm transitions are associated with conformational changes involving Chls *a*/*c* and carotenoid's coupling and the significantly reduced population of red-Fxs operates as a switch between light harvesting and photoprotective states. Such changes are expected to affect the excitation delocalization/localization on the pigments that contribute to the functionality of the photosynthetic complexes.^24^

## 2. Experimental

### 2.1 Sample preparation

The culture of *Thalassiosira pseudonana* (CCAP1085/12) was obtained from CCAP (Culture Collection of Algae and Protozoa). Cultures were grown in f/2-Si medium, pH 8, at 19 °C, with 12 h:12 h dark–light cycle under white light. The light intensity used in the growth of the cell cultures was provided by LEDs and it was either 15 μmol photons per m^2^ per s, 150 μmol photons per m^2^ per s or 350 μmol photons per m^2^ per s. Cells were harvested in exponential phase and concentrated using centrifugation at 5000 rpm for 3 minutes.

### 2.2 Absorption measurements

Absorption spectra of the cells in H_2_O with OD 0.2 at 674 nm were recorded in a 3 mm cuvette by a Cary 60 UV-Vis spectrometer (Agilent Technologies, USA).

### 2.3 Fluorescence measurements

The Cary Eclipse Fluorescence Spectrophotometer (Agilent Technologies, USA) was used for recording the fluorescent spectra of the samples in a 3 mm cuvette. The slit widths for both the excitation and emission monochromators were set at 5.0 nm. The samples used for the measurements had an OD 0.2 at 674 nm.

### 2.4 Raman measurements

Raman data were collected by a confocal LabRAM (HORIBA Jobin Yvon, Kyoto, Japan) equipped with a CCD detector and 1800 grooves per mm grating. The spectral resolution was 5 cm^−1^. It is equipped with an Olympus BX41 microscope. The 442 nm excitation laser beam was provided by KIMMON He–Cd laser, and the laser power incident on the sample was 10 mW. The total accumulation time for each measurement was 1 min. Every spectrum is the average of 40 measurements. The samples used for the measurements had an OD 0.2 at 674 nm. The samples were placed in a quartz cuvette (1 cm) and the laser beam was defocused on the sample (50 microliters) to avoid sample damage. Under these conditions the sample was very stable for 60 s. Uv-vis spectra were taken after 60 s exposure to check for sample stability. Different samples from cells of the same concentration were accumulated for 60 s each resulting in a total accumulation time of 40 min. All the experiments were repeated three times from different *Thalassiosira pseudonana* (CCAP1085/12) cultures and the results were highly reproducible.

## 3. Results and discussion

### 3.1 Optical absorption of *T. pseudonana* cells

**Fig. 1:**
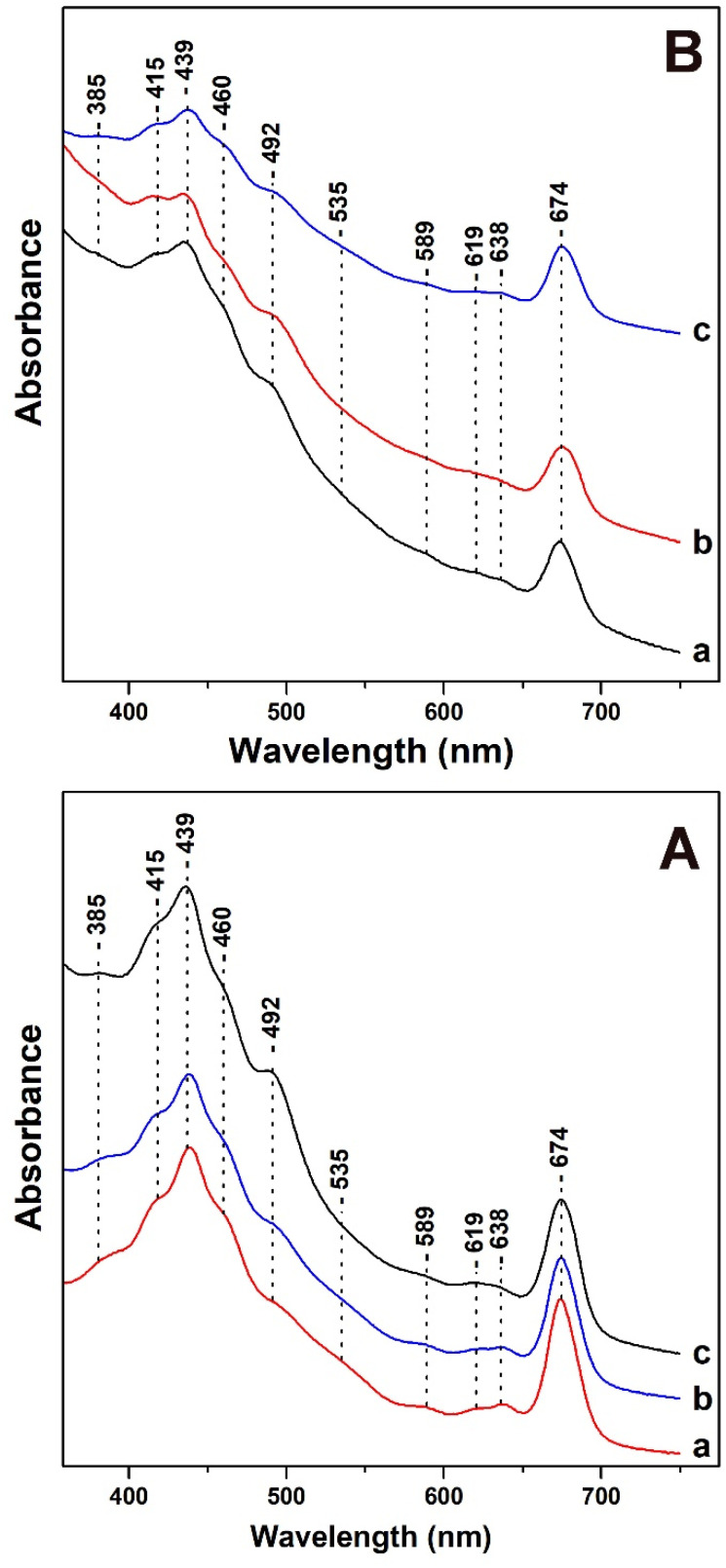
Uv-vis absorption spectra of *T. pseudonana* cells grown under: panel A (a) 15 μmol photons per m^2^ per s (b) 150 μmol photons per m^2^ per s (c) 350 μmol photons per m^2^ per s white light. Panel B (a) 350 μmol photons per m^2^ per s for four days (b) 350 μmol photons per m^2^ per s for nine days (c) 350 μmol photons per m^2^ per s for four days followed by exposure to 15 μmol photons per m^2^ per s for five days (white light).

In Fig. 1, panels A and B, we present the Uv-vis absorption spectra of *T. pseudonana* cells grown under variable light intensity of white light from measurements of three different samples. All samples used in Fig. 1 had similar OD values (optical density) measured at 674 nm.

In panel A, trace a, under LL cell growth conditions of 15 μmol photons per m^2^ per s the bands in the 385–460 nm Soret region are due to Chls *a*/*c* and carotenoids. The 492 nm band has contributions from blue-shifted Fxs and Dds and the broad shoulder in the 500–560 nm range has been attributed to red-shifted Fxs. The transitions at 439 (Soret), 619 (Q_*x*_) and 674 (Q_*y*_) nm are attributed to Chl *a* and at 460 (Soret), 589 (Q_*x*_) and 638 (Q_*y*_) nm to Chl *c*.^12,16,35^ There are no differences in the absorption spectrum shown in trace b of the cells grown under IL. A noticeable increase in the 492 nm band with a concomitant decrease in the broad shoulder in the 500–560 nm spectral region shown in trace c is occurred upon increasing the light intensity of the cell-growth conditions to HL. In addition, there no changes in the Q_*x*_ and Q_*y*_ bands of Chl *c*. In panel B, traces a and b were obtained with HL growth for four days (trace a) and for nine days (trace b). In panel B, trace c was obtained from cells grown under HL for four days and subsequently under LL for five days. Traces a and b are similar to those obtained in panel A with the exception of (a) a slight increase in the band at 492 nm indicating that the longer exposure to HL affects the 492 nm band, and (b) a decrease in intensity in the 380–480 nm region.

It should be noted that the intensity of the Q_*x*_ and Q_*y*_ band of Chl *c* remained unaffected. We attribute the decrease in the 380–480 nm region to the decrease in the content of both blue- and red-shifted Fxs. In trace c, the initial incubation of the cells to HL intensity, which is followed by incubation to LL intensity shows a decrease in the intensity of the 492 nm band, increase in the 460 nm band and in the shoulder at 500–560 nm. This observation indicates a reversible light-intensity behavior of Dds and Fxs composition in the cells. All the experiments presented in Fig. 1 were repeated three times from different cultures of *T. pseudonana* and the results were highly reproducible.

### 3.2 Resonance Raman of Chl *a*/*c* and Fx/Dd/Dt in *T. pseudonana* cells

Four marker bands in the resonance Raman spectra of carotenoids provide valuable information regarding their configuration and conformation.^27,28,33–37^ The frequencies of the marker Raman bands of carotenoids and Chl *a*/*c* as well their position and amplitude changes under the different lighting conditions is shown in ESI.† A comparison between different runs of measurements in the Raman marker bands under the different growing conditions, is also presented in ESI.† The *ν*_1_ which arise from the C=C stretching vibration, the *ν*_2_ which arise from the C–C stretch coupled with the C–H in plane bending modes, the *ν*_3_ which arise from the in-plane rocking vibrations of the methyl groups attached to the conjugated chain, coupled with in plane bending modes of the adjacent C–H's. The *ν*_3_ is also a fingerprint of the conjugated end cycle conformation.^34^ Finally, the *ν*_4_ which arise from the C–H out of plane wagging motions coupled with the C–C torsional modes (out-of-plane twists of the carbon backbone).^28^ Of the major differences observed in the Raman spectra between Fx, Dd and Dt are (1) the very intense *ν*_2_ of Dt observed at 1159 cm^−1^ and (2) the appearance of the 1183 cm^−1^ mode in Fx but not in the spectra of neither the Dd nor the Dt.^28,33–35^ In the region of *ν*_3_ there are also noticeable differences between Fx/Dd/Dt. Of note is the intense 1011 cm^−1^ mode present in Dt and not in Fx/Dd. The 442 nm excitation resonance Raman spectra of Chl *a*/*c* contain vibrational modes sensitive to the conformations of the Chls *a*/*c* macrocycles. The large number of Chls *a*/*c* in the cells makes it difficult of making detailed analysis on the dynamic behavior of the individual Chls *a*/*c* in the cells. Taken together, Raman excitation at 442 nm can provide significant information for all the Fx/Dd/Dt and Chl *a*/*c* pigments in the *T. pseudonana* cells and probe their structure–function relationship and possible conformational–structural pigment content changes they might exhibit under different light conditions at room temperature.

**Fig. 2:**
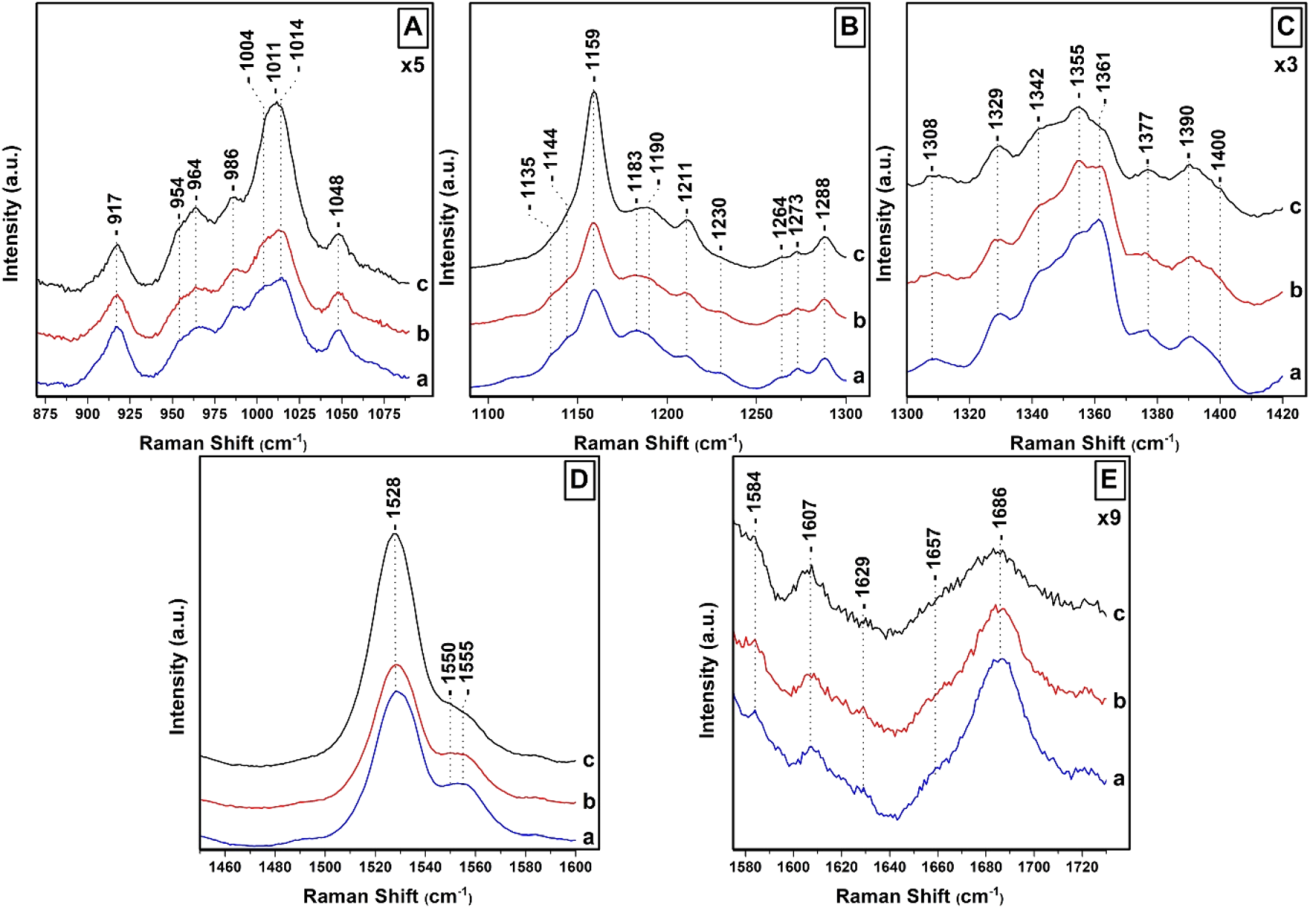
Raman spectra of *T. pseudonana* cells grown under white light 15 μmol photons per m^2^ per s (traces a), 150 μmol photons per m^2^ per s (traces b) and 350 μmol photons per m^2^ per s (traces c). The 442 nm excitation laser beam was provided by KIMMON He–Cd laser and the laser power incident on the sample was 10 mW. The total accumulation time for each measurement was 1 min. Every spectrum is the average of 40 measurements.

Fig. 2 shows the 442 nm excitation Raman spectra of *T. pseudonana* cells grown under white light of 15 μmol photons per m^2^ per s (traces a), 150 μmol photons per m^2^ per s (traces b) and 350 μmol photons per m^2^ per s (traces c) from measurements of three different samples. Excitation within the 442 nm laser line produces a spectrum dominated by totally symmetric Franck–Condon-active modes aligned along the *x*-axis of the Chls *a*/*c* macrocycles. In panel A, trace a, the spectral 850–1050 cm^−1^ range is characterized by peaks at 964 cm^−1^ due to *ν*_4_ of Fx, 986 cm^−1^ due to *ν*_4_ of Dd, and 1004/1014 cm^−1^ due to *ν*_3_ of Fx/Dd.^27,37–39^ The peaks at 917, 954 and 1048 cm^−1^ originate from Chl *a* and have similar intensities in traces a–c. There are minor fluctuations in the intensities of the 964 cm^−1^ peak shown in traces a–c but significant changes in the intensities of the 1004/1014 cm^−1^ peaks shown in trace c. Of note, is the presence of the intense broad peak centered at 1011 cm^−1^ shown in trace c that we assign to the *ν*_3_ of Dts and provides evidence for the conversion of Dds to Dts in the cells which is the major photoprotection mechanism in diatoms and is active under the present room temperature experimental conditions. In panel B (traces a–c), the *ν*_2_ spectral region contains marker bands for Fxs which are characterized by peaks at 1159, 1183, 1211 and 1230 cm^−1^ whereas the 1190 and 1211 cm^−1^ are due to the presence of Dds.^27,28^ The increased intensity of the 1159 and 1211 cm^−1^ peaks in trace c indicates that both peaks also have contributions from Dds and Dts and also that the C–C mode adopts a conformational distortion under HL conditions. In trace c, the 1183 and 1230 cm^−1^ peaks have lost intensity, as expected under HL conditions, providing further evidence that they are mainly marker bands for Fxs. The C–H rocking is the internal coordinate that dominates the modes between 1250 and 1400 cm^−1^. The 1135 and 1144 cm^−1^ peaks are due to Chl *a*/*c*. The *ν*(C_a_N) bands in the 1300–1400 cm^−1^ region are highly resolved in the 442 nm spectrum. In panel C, the RR spectra in the 1300–1420 cm^−1^ spectral region are similar to those reported previously and are consistent with Franck–Condon activity of in-plane ring modes in resonance with the strongly allowed B_*x*_ transitions of Chl *a*/*c*.^27^ In trace a, the peaks at 1329, 1342 and 1377 cm^−1^ originate from the *ν*(C_a_N) of five- and six-coordinated Chl *a* and the peaks at 1355 and 1361 cm^−1^ from the *ν*(C_a_N) of Chl *c*. All Chl *a* peaks have similar intensities in traces b and c. On the other hand, we attribute the intensity increase of the 1355 cm^−1^ and concomitant decrease of the 1361 cm^−1^ peaks to conformational changes of the Chl *c* macrocycle resulting in the intensity/frequency changes observed in traces b and c.^27^ The conformational changes in the macrocycle of Chl *c* can be used in studies for the location and conformational dynamics of protein-bound Chl *c* in the membranes of diatoms. In panel D, trace a, the *ν*_1_ of Fxs is observed at 1528 cm^−1^ and the peak at 1555 cm^−1^ has contributions from the *ν*(C_a_C_b_) of Chls *a*/*c*. There are no noticeable changes in trace b but in trace c there is a substantial increase in the *ν*_1_ that we attribute to the presence of Dds/Dts which have an intense *ν*_1_. Of particular interest is the change in the band shape and intensity of the Chls *a*/*c* peaks at 1555 cm^−1^ shown in trace c. Finally, in panel E the 1607 cm^−1^ peak arise from the methine bridges of Chls *a*/*c*, the frequency of which depends on the coordination state of the central Mg^2+^ ion and the 1629 cm^−1^ from the stretching mode of the conjugated vinyl group in position C_2_.^27,37^ The methine bridge mode is observed at approximately 1600 cm^−1^ when Mg^2+^ is six-coordinated and slightly distorted by its protein environment and is up-shifted to 1612–1615 cm^−1^ for five-coordinated Mg^2+^.^37^ The 1607 cm^−1^ band it could arise either from a five-coordinated Mg^2+^ with an unusually planar conjugated system or from a six-coordinated Mg^2+^ distorted by the protein environment. When Mg^2+^ interacts with a histidine residue, the methine bridge is observed in the 1612–1615 cm^−1^ frequency range. The 1607 cm^−1^ frequency we have observed makes it unlikely that Chl *a*/*c* interacts with such a strong ligand. The coordination sensitive modes in Chl *a*/*c* are localized at 1555 and 1607 cm^−1^ indicating that most Chl *a*/*c* molecules are five-coordinated or there certain six-coordinated Chls *a*/*c* molecules with weak axial ligands. It is clear that in panel D, trace c there is a substantial decrease of the 1555 cm^−1^ peak and an additional weak shoulder on the low frequency shoulder at 1550 cm^−1^ without any significant change in the frequency of the 1607 cm^−1^ band. This shift may be indicative of a change in the orientation of certain Chl *a*/*c* with respect to Dd/Dt in the cells, or that the macrocycle angles between the pyrrole rings of certain Chl *a*/*c* are experienced distortion in the cells and/or a conformational change in the macrocycles, or some combination of these three possibilities. The observation of the 1355/1361 cm^−1^ of Chl *c* shown in panel C, trace c, clearly demonstrate that there is a subset of Chl *c* molecules that they experience changes in the core size.

In general, the vibrational modes containing bonds that are parallel to the electronic transition dipole moment and are distorted in the excited state are expected to undergo the strongest enhancement. The carbonyl stretching modes of the conjugated C=O groups are located at 1657 cm^−1^ due to Fx and in the 1670–1700 cm^−1^ region due to *ν*(C_13_=O) of Chl *a*/*c*.^27,35^ The *ν*(C_13_=O) in a free form of interactions environment is located at 1700 cm^−1^ and is down-shifted near 1690 cm^−1^ in a polar environment; there is a further decrease, depending of the strength of the interaction near 1660 cm^−1^ when this group is involved in H-bonding interactions. In case the C=O is present in a polar environment the *ν*(C_13_=O) is expected near 1680–1685 cm^−1^. The frequency of the 13-keto carbonyl mode is extremely sensitive to the environment of this group, therefore the binding sites of all Chl *a* molecules share very similar physicochemical properties. The very broad peak centered at 1686 cm^−1^ is indicative of many orientations and/or interactions with nearby residues of the C=O of Chl *a*/*c*. In trace b, there is a decrease in the intensity of the 1686 cm^−1^ peak and in trace c both the 1657 and 1686 cm^−1^ modes exhibit substantial reduced peak intensities. These observations indicate reduced contribution from Fxs and conformational changes of the Chl *a*/*c* carbonyl groups as result of the pigment redistribution, and thus, with their interactions with the C=O group. In trace c, there is substantial decrease in the intensity of the carbonyl modes in the 1670–1700 cm^−1^ spectral region that we attribute to conformational changes in the environment of the C=O of Chls *c*. Therefore, under HL growth conditions a sub-population of Chls *c* exhibit intermolecular interactions and structural changes of the interacting pigment environments quite different from those in the cells grown under LL or IL conditions.

**Fig. 3:**
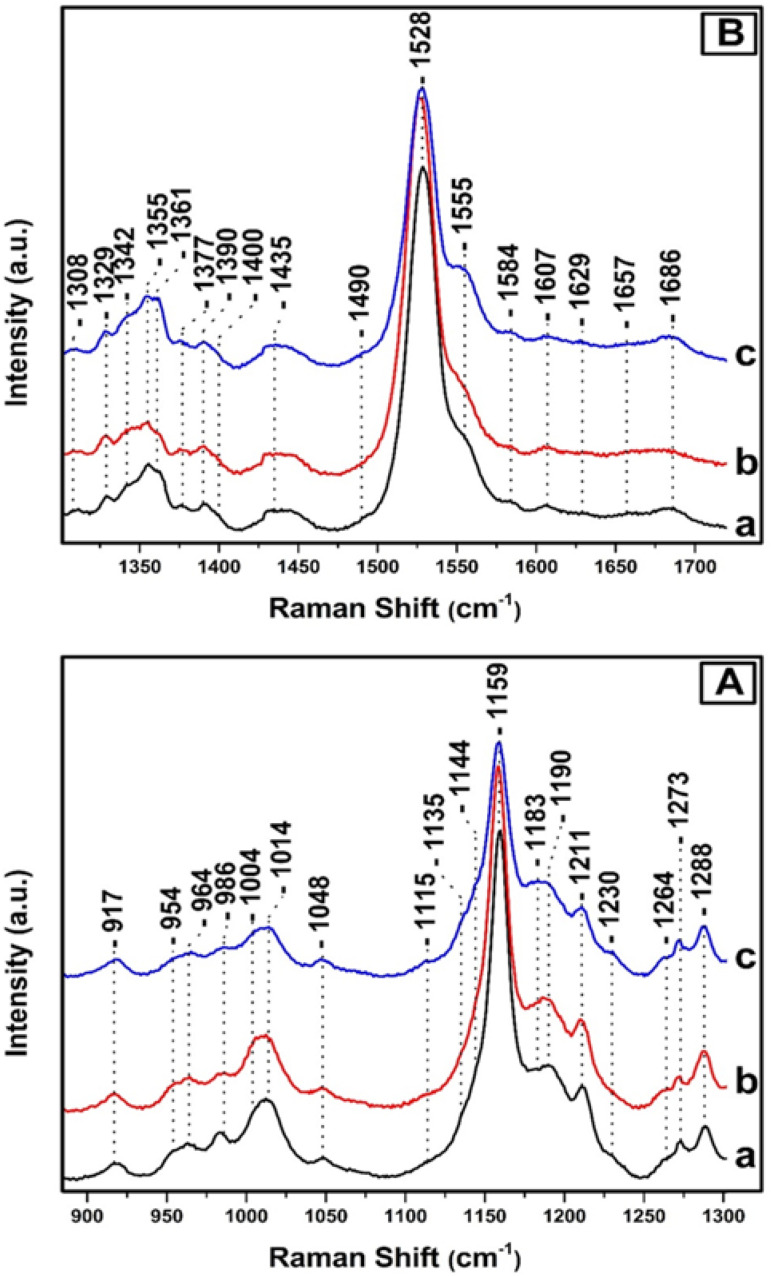
Raman spectra of *T. pseudonana* cells grown under 350 μmol photons per m^2^ per s for four days (trace a), 350 μmol photons per m^2^ per s for nine days (trace b) and 350 μmol photons per m^2^ per s for four days and subsequently in 15 μmol photons per m^2^ per s for five days (trace c). The 442 nm excitation laser beam was provided by KIMMON He–Cd laser and the laser power incident on the sample was 10 mW. The total accumulation time for each measurement was 1 min. Every spectrum is the average of 40 measurements.

Fig. 3, depicts the Raman spectra of *T. pseudonana* cells grown under white light of 350 μmol photons per m^2^ per s for four days (trace a), 350 μmol photons per m^2^ per s for nine days (trace b), and 350 μmol photons per m^2^ per s for four days, followed by five days growth period of 15 μmol photons per m^2^ per s (trace c). All spectra were obtained from measurements of three different samples. In panels A and B, the intensity and frequencies of peaks in traces a and b are similar to those presented in Fig. 2, trace c. Therefore, there are no significant differences in the pigment content after extending the growth period from four to nine days under 350 μmol photons per m^2^ per s of white light. Comparison of traces a/b and c in panels A and B reveals significant differences in the peak intensities of the major marker bands of Fxs/Dds/Dts and Chl *a*/*c*. In particular, in panel A, trace c the *ν*_4_ of Dd at 986 cm^−1^, *ν*_3_ of Dt at 1011 cm^−1^, and *ν*_2_ at 1159 cm^−1^ have lost significant intensity. In the *ν*_3_ region there are two peaks at 1004 and 1014 cm^−1^ as they were observed in trace a, Fig. 2 and assigned to Fx and Dd, respectively. Importantly, there is an increase in the intensity of the 1183 cm^−1^ peak which is due to Fxs and a significant decrease in the *ν*_2_ at 1159 which has also contributions from Dd/Dt. Overall the spectrum in trace c resembles that of trace a in Fig. 2 that was obtained from samples grown under LL intensity. This observation indicates that in trace c the number of Dts pigments has decreased and those of Fx increased demonstrating the reversibility of the photoprotection mechanism that involves Dd/Dt in the cells. In panel B, traces a and b, the peaks at 1329, 1344, and 1379 cm^−1^ originate from the *ν*(C_a_N) of five- and six-coordinated Chl *a*, and those at 1355 and 1361 cm^−1^ from the *ν*(C_a_N) of Chl *c*. The peak at 1555 cm^−1^ that has contributions from the *ν*(C_a_C_b_) of Chls *a*/*c* show high similarity with those in the spectrum shown in Fig. 2, panel C, trace c, demonstrating that there are not any changes in the Chl *a*/*c* content or in their dynamic behavior due to the different exposure time to HL. The rest of the peak intensities of the major bands in trace a and b resembles those obtained under HL growth condition presented in trace c, Fig. 2. In trace c, Fig. 3, panel B, there is a change in the behavior of the *ν*_4_ of the two conformations of Chl *c* at 1355 and 1361 cm^−1^ and the *ν*(C_a_C_b_) at 1555 cm^−1^ as compared to that obtained in traces a and b. The higher frequency conformer of Chl *c* has gained intensity whereas the lower frequency conformer has lost intensity in a similar behavior to that obtained in the spectra of the samples grown under LL conditions. The *ν*_4_ marker bands of Chl *c* in conjunction with the *ν*(C_a_C_b_) are indicators of the planarity of the heme or ruffling show the same behavior as presented in Fig. 2, traces a and b. Therefore, Chl *c* is present in a reversible LL–HL dependent conformations characteristic of a planar and nonplanar configuration of the porphyrin macrocycle. The rest of the peak intensities of the major bands in trace c resembles those obtained under LL growth condition presented in Fig. 2, trace a. The ability of the cell to regenerate the pigment content with similar dynamic structure–function relationship indicates the reversible light-intensity dependency of diatoms.

**Fig. 4:**
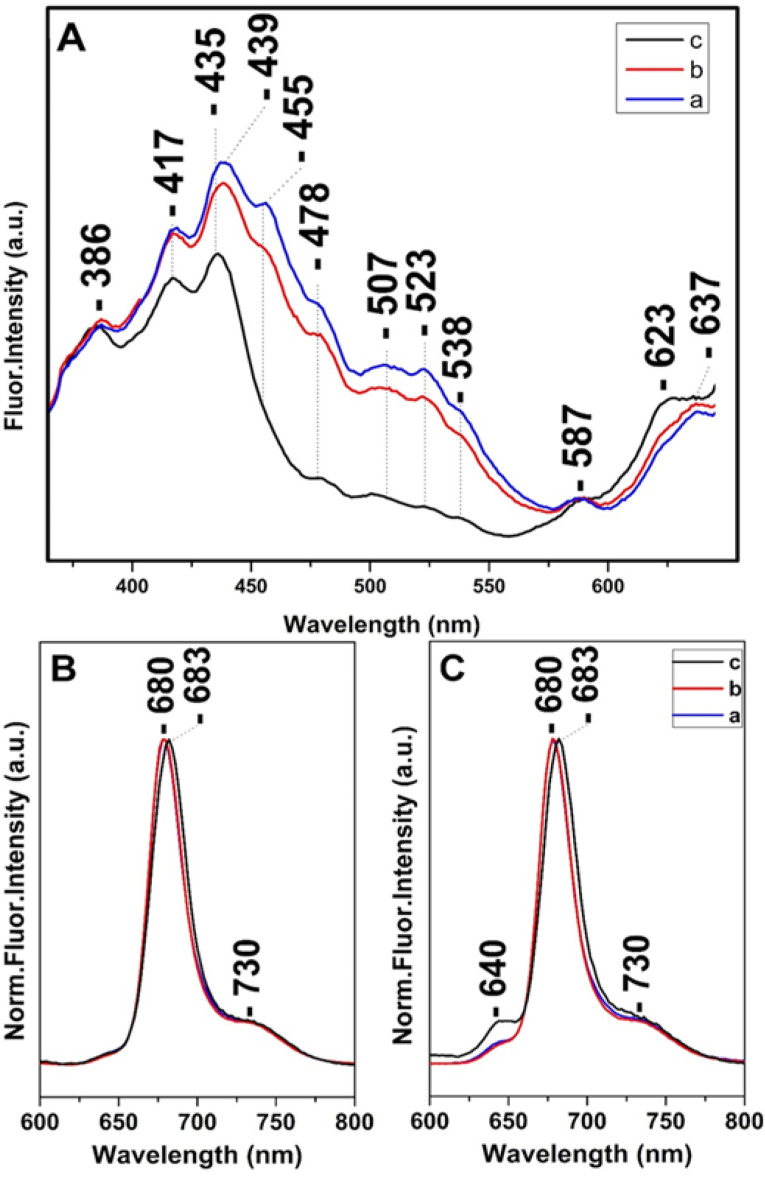
(Panel A) Room temperature fluorescence excitation spectra at 680 nm of *T. pseudonana* cells grown under 15 μmol photons per m^2^ per s (trace a), 150 μmol photons per m^2^ per s (trace b) and 350 μmol photons per m^2^ per s (trace c). Room temperature normalized fluorescence emission spectra excited at 418 nm (Panel B) and 460 nm (Panel C) of *T. pseudonana* cells grown under white light of 15 μmol photons per m^2^ per s (trace a), 150 μmol photons per m^2^ per s (trace b) and 350 μmol photons per m^2^ per s (trace c).

Steady state and time-resolved fluorescence studies have been employed towards the elucidation of the chemical dynamic processes involved in diatoms.^12,13,30,38–40^Fig. 4 shows in panel A the fluorescence-excitation spectra at 680 nm of *T. pseudonana* cells grown under white light of 15 μmol photons per m^2^ per s (trace a), 150 μmol photons per m^2^ per s (trace b) and 350 μmol photons per m^2^ per s (trace c) from measurements of three different samples, measured at room temperature. Traces a and b show good agreement with the absorptivity shown in Fig. 1 and demonstrate that there is sufficient energy transfer to Chls *a* when Chls *c* and Fxs were excited in the 400–550 nm range indicating similar energy pathways to Chl *a* as previously reported. The ratio of Chl *c* to Chl *a* and Fx to Chl *a* varied very little with growth irradiance in the range from 15 to 150 μmol photons per m^2^ per s and both blue and red-shifted Fx and Chl *c* are able to transfer absorbed light energy to Chl *a*. In combination with the Raman data the close similarity in the spectra proves that the pigment content and their dynamic behavior is similar when the cells are cultured under LL and IL conditions. In trace c the 507, 523 and 538 nm peaks are absent, whereas that at 455 nm has retained less than 1/3 of its intensity shown as a broad tailing absorption. This observation indicates that the energy transfer to Chls *a* under HL conditions does not involve the red-shifted Fxs that absorb in the 450–550 nm range. The data are in accord with the absorption data presented in Fig. 1 and the Raman data shown in Fig. 2 from samples grown under HL conditions. In trace c there is also a blue-shifted transition from 439 to 435 nm and the ratio of the 435/417 nm peaks has increased relative to that observed in traces a and b. We attribute the 435/417 nm peaks to blue-shifted Fx and Chl *a* that are present under HL growth conditions. Of note is the relatively small increase at 587 nm and at 637 nm when the Chl *c* (Q_*x*_) and Chl *a* (Q_*x*_) were excited, respectively. Panels B and C depict the normalized room temperature fluorescence emission spectra of *T. pseudonana* cells grown under white light of 15 μmol photons per m^2^ per s (trace a), 150 μmol photons per m^2^ per s (trace b) and 350 μmol photons per m^2^ per s (trace c) excited at 418 nm (panel B) and 460 nm (panel C). The 418 and 460 nm excitation lights can be absorbed by Chls *a* and Chls *c*, respectively. The 418 nm excitation showed a peak at 680 nm (traces a and b) which originated from Chls *a*.^12,16,17,38^ In trace c the emission fluorescence has slightly red-shifted to 683 nm suggesting that Chls *a* exhibits different energy forms under LL and HL intensity growth conditions ranging from 680 to 683 nm. In panel C, the 460 nm excitation showed a small additional peak at 640 nm (traces a and b) which originate from Chls *c*; the intensity ratio of *I*_640_/*I*_680_ is 0.05 in traces a and b and 0.13 in trace c. This observation clearly demonstrates that the energy transfer from Chls *c* to Chls *a* is less efficient under HL conditions (trace c). We propose that the blue-shifted from 439 to 435 nm and red-shifted from 680 to 683 nm transitions originate from a light-dependent fluctuating environment due to structural reorganization of the pigment–protein complexes that affects the energy distribution in diatoms.

The regulation of the excitation energy transfer process/pathway in the cells of diatoms induced by the HL growth conditions and the observed reversibility of the process when the cells are exposed from HL to LL is important for diatoms to adapt fluctuating light intensity in their living environments. In the investigation of *T. pseudonana* cells we are not concerned about the variation of the pigment content under gentle or harsher FCPs sample preparation and the loss of peripheral pigments during the purification procedures. On this line, it should be noted that none the FCPs from the centric diatom *Chaetoceros gracilis* display similar stoichiometry with that observed in the pennate diatom *Phaeodactylum tricornutum*.^28^ Furthermore, even if the obtained structures of FCPs from different pennate or centric diatoms are similar in terms of the polypeptide structure and pigment organization, they differ by the stoichiometry of the bound pigments. Therefore, reliable conclusions can be reached about the functionality and dynamics of a specific diatom only if there is a comparison of the structure/function relationship of the FCPs and the cells from the same diatom. Although there are significant differences in the ratio of the pigment composition of different diatoms there is consensus on the energy transfer pathways involving Fxs/Dds and Chls *c* to Chls *a* energy transfer but not on the energy transfer efficiency. Variations in the composition of Cars are regulated through the DD cycle and interact with photo-protective light harvesting complexes protein isoforms to lower the efficiency of the energy transfer from antennae pigments to RC.^21–24^

We have observed and characterized two reversible light dependent energy transfer pathways observed under LL and HL conditions that are discussed based on the conformational and structural modifications of the major marker bands of Chls *a*/*c*, Fxs/Dds/Dts detected under the same experimental conditions in conjunction with the fluorescence-excitation experiments. A cascade of events including orientation changes of Chl *a*/*c* coupled with structural changes of Fxs/Dds/Dts has been identified. The photoregulation of the pigment content and their functionalities plays a role in the switching mechanism between light-harvesting and photoprotection functions as well in varying excitonic energy level populations. In the absence of a crystal structure of *T. pseudonana* the Raman data are very informative with respect to the coordination and possible conformations of Chl *a*/*c*. The data are compatible with the presence of five-coordinated Mg^2+^ with an unusually planar conjugated system and from six-coordinated Mg^2+^ with weak axial ligands distorted by the protein environment. Based on the behavior of the marker bands under LL and HL conditions we suggest that there are changes in the orientation of certain Chl *a*/*c* with respect to Fx/Dd/Dt in the cells and macrocycle angles changes between the pyrrole rings of certain Chl *a*/*c* that experience distortion and/or conformational changes. As a result, the overall pigment rearrangement and composition of the binding sites is expected to have a local effect on the excited state energy landscape and, thus, on the efficiency. This finds support by the fact that the blue-shifted 439/435 nm and red-shifted 680/683 nm transitions exist in the cells under HL.

Under LL and IL intensity cell growth conditions all Fxs and Chls *c* contribute to the energy transfer to Chls *a* in *T. pseudonana* cells. Upon excitation at 460 nm of HL intensity cell-growth, photons were absorbed by Chls *c*, and the higher-energy Fxs and Dds. In addition, the Q_*y*_ emission of Chls *c* increased and the ratio of *I*_640_/*I*_680_ raised from 0.05 to 0.13 indicating that the excitation energy is transferred with a reduced energy transfer efficiency from Chls *c* to Chls *a*. The energy transfer from Chls *c* to Chls *a* is accompanied by structural changes of Chls *c* having characteristic *ν*_4_ frequencies at 1355/1361 cm^−1^. These results indicate that *T. pseudonana* switch its function from light-harvesting to energy-quenching *via* arrangements of the energy-transfer pathways that do not involve the red-shifted Fxs under HL growth conditions. We propose that the light-dependent conformational changes of Chl *c* and the absence of the red-shifted Fxs are the key contributors that affect the excitation localization/delocalization that contribute to the emission.

In the absence of energy transfer from the red-shifted Fxs under HL conditions, the blue-shifted to red-shifted Fxs energy transfer pathway, as previously reported, is not operative in *T. pseudonana*.^41^ The blue-shifted Fxs and Dds coupled with Chls *c* are the determinant factors in the energy transfer pathway in cells grown under HL conditions. It should be noted that under LL and IL cell growth conditions both the lower and higher energy Fxs are involved in the energy transfer to Chls *a*. In the absence of the red-shifted Fxs, upon excitation at 457 nm, photons are absorbed by Chls *c* and by the blue-shifted Fxs. Therefore, the excitation energy is transferred from the blue-shifted Fxs to Chls *a*. These differences play an important harvesting role under water where the useful to Chls *a* red light is minimized and higher energy photons arrive. Taken together, we suggest that under HL growth conditions a sub-population of Chls *c* exhibit intermolecular interactions quite different from those in the cells grown under LL and/or IL conditions. Recently, structural changes of the pigments environment in the FCP from *P. tricornutum* were invoked to explain the pH effect on the switch of the FCP from light-harvesting to energy-quenching *via* modification of the energy-transfer pathways.^42^

## 4. Conclusions

A reversible light-intensity behavior of Dds and Fxs composition in the cells of *T. pseudonana* was observed. The observed LL to HL reversible transitions are accompanied by structural modifications of Chls *a*/*c* and the lack of the red-shifted Fxs. The ability of *T. pseudonana* to regenerate the red-shifted Fxs and to restore the initial Chls *a*/*c* conformations is of profound importance towards our understanding of the factors involved in the function of diatoms from light-harvesting to energy-quenching photosynthetic biomachines.

## Author contributions

CT performed the experiments and analyzed the results and CV analyzed the results and wrote the paper.

## Conflicts of interest

There are no conflicts to declare.

## Supplementary Material

RA-012-D2RA05284A-s001
