# Supplementary material for: Evidence for reversible light-dependent transitions in the photosynthetic pigments of diatoms
Source: RSC Adv. 2022 Nov 3;12(49):31555–63. doi: 10.1039/d2ra05284a (PMC9631684; doi:10.1039/d2ra05284a)
Supplement: RA-012-D2RA05284A-s001 [file RA-012-D2RA05284A-s001.pdf]

Electronic Supplementary Material (ESI) for RSC Advances.
This journal is © The Royal Society of Chemistry 2022

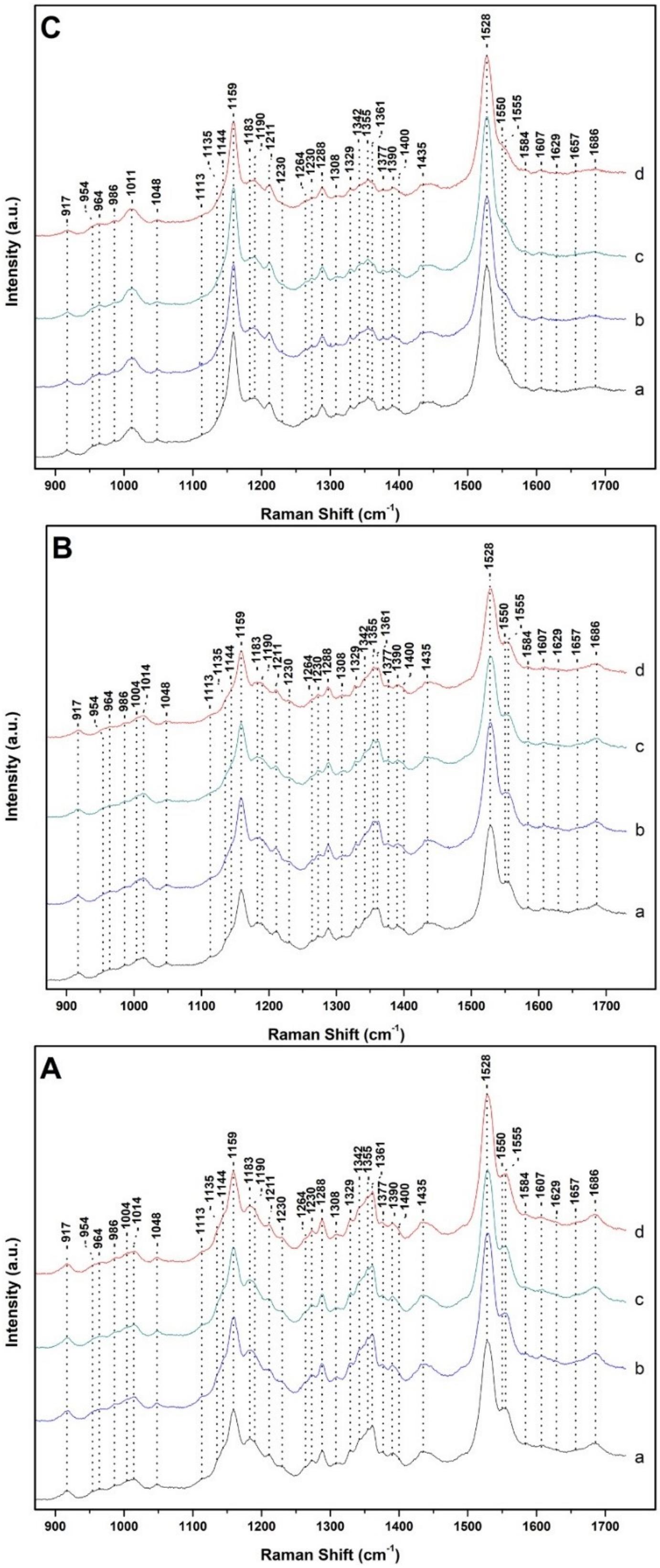

SI Figure 1. Resonance Raman spectra of the Low-light (LL, Panel A), Intermediate Light (IL, Panel B) and High Light (HL, Panels C). In each panel, spectra a ,b, c, and d correspond to a 10 min spectrum resulted from ten individual spectra of 60 sec each; total accumulation 10

min. The sum of the four spectra (a+b+c+d) in each panel are the spectra of the 40 min presented in the paper.

Table SI. Raman Frequencies of the marker bands of Fx, Dd, Chl *a* and Chl *c* under IL, LL and HL growth conditions. (+) indicates an increase in the intensity and (-) a decrease in the intensity.

	Band Position (cm^{-1})		
	LL	IL	HL
Fx (v_1)	1528	1528	1528
Fx (v_2)	1159	1159	1159
Fx (v_3)	1004/1014	1004/1014	1004/1014(-)
Fx (v_4)	964	964	964
Dd/Dt (v_1)	1528	1528	1528(+)
Dd/Dt(v_2)	1159	1159	1159(+)
Dd/Dt(v_3)	1011	1011	1011(+)
Dd/Dt(v_4)	986	986	986
Chl a(C_aN)	1342	1342	1342(-)
Chl a(C_aN)	1377	1377	1377(-)
Chl c(C_aN)	1355	1355	1355(-)
Chl c(C_aN)	1361	1361(-)	1361(-)
